# Supplementary material for: Patterns of Intron Gain and Loss in Fungi
Source: PLoS Biol. 2004 Nov 30;2(12):e422. doi: 10.1371/journal.pbio.0020422 (PMC532390; doi:10.1371/journal.pbio.0020422)
Supplement: Table S1 — Also available at http://genes.mit.edu/NielsenEtAl/. (4.3 MB ZIP). [file pbio.0020422.st001.zip › NielsenEtAl/html/210.html]

AN8959.1.NCU02801.1.MG01585.1.FG01031.1


```
 CLUSTAL W (1.82) Multiple Sequence Alignments - Introns Inserted


Sequence 1: MG01585.1	215 aa
Sequence 2: FG01031.1	186 aa
Sequence 3: NCU02801.1	209 aa
Sequence 4: AN8959.1	180 aa
Alignment Length: 236 aa
Number Identitical Residues: 52 aa
Alignment Score (without introns) 3373


MG01585.1 	MTTYIPQITLPYSVFTSPAASILLPVGLGSLVGWSTARKPPSPILSSILLLLCASQLTWT
NCU02801.1	MTSYIPQLTLPSSIFTSAPASILLPVALGTAVGFGTG-----------------------
FG01031.1 	MTTYIPALTLPDSVFLKPALSVLLPIGLGTAVGFS-------------------------
AN8959.1  	MP---WSFVLPQAIFASPLLATLTPVATGSTIGYLVN-----------------------
          	*.     :.** ::* ..  : * *:. *: :*:                          

MG01585.1 	YHKAA~KK~TKAKYAALKQPPYSPPAWVFGPAWTVLYG~LMGYAAYRAVDTGMSP-----
NCU02801.1	---FV1SD~QKRQYHSLRQPPLRPPSSVFGPVWTLLYG~TMGYAAHRAYLFGTSSSSSPS
FG01031.1 	----A~TQ1TQKTYLALKKPSVQPPPWLFGPVWTILYG2VMGYAAYRVADIGLSP-----
AN8959.1  	----R1YG~TKPKYHALKQPPASPPSWLFPPVWTLLYG~LTGYASYHFTVNTLEP-----
          	          :  * :*::*.  **. :* *.**:***   ***:::      ..     

MG01585.1 	--LSSVIHQRMTKHGATLYSIQLGLNLVWMPLFFGLKRPVEASVDILALVG-INSYLTYL
NCU02801.1	LLLSNPSVVPTTYHTATLYTIQLGLNLIWMPLFFGLRRPILATLDCAALVG-INAYLAWT
FG01031.1 	--FSSPETIVNTRQAMTVYSIQLGLNLIWTPLFFGLYRPIEASVDIITLLG-VNGYLTYL
AN8959.1  	----------DTTITQTLYTAQLFLNHLWMPLFFAARKPILAAGDIVLLGGTVATLMREL
          	           *    *:*: ** ** :* ****.  :*: *: *   * *::   :   

MG01585.1 	WGS-VDRVAGLCLAPYVAWLSYASYLCIGAGYLNGWDIKDKETPKQQ------------
NCU02801.1	WGTQIDAVAGWLMVPYVCWLGFATYLSAGSGYLNEWDFSEERIRKGEEEQQKRKGEKGL
FG01031.1 	YSS-IDSTAAWCQVPYLGWLTFATYLCTTIGHLNNWDLASGEINKKE------------
AN8959.1  	WGS--DRVSFWLFAPYAAWLGYATYLNFGTGILNNWRIPDVPEERKRRNE---------
          	:.:  * .:    .**  ** :*:**    * ** * : .    : . ..
```
